# A Mixture-Function Mortality Model: Illustration of the Evolution of Premature Mortality

**DOI:** 10.1007/s10680-019-09552-x

**Published:** 2020-03-20

**Authors:** Lucia Zanotto, Vladimir Canudas-Romo, Stefano Mazzuco

**Affiliations:** 1 Department of Economics, Ca’ Foscari University of Venice, Venice, Italy; 2 School of Demography, Australian National University, Canberra, Australia; 3 Department of Statistical Sciences, University of Padua, Padua, Italy

**Keywords:** Mortality model, Mixture distribution, Skew Normal distribution, Premature mortality, Life expectancy

## Abstract

Premature mortality is often a neglected component of overall deaths, and the most difficult to identify. However, it is important to estimate its prevalence. Following Pearson’s theory about mortality components, a definition of premature deaths and a parametric model to study its transformations are introduced. The model is a mixture of three distributions: a Half Normal for the first part of the death curve and two Skew Normals to fit the remaining pieces. One advantage of the model is the possibility of obtaining an explicit equation to compute life expectancy at birth and to break it down into mortality components. We estimated the mixture model for Sweden, France, East Germany and Czech Republic. In addition, to the well-known reduction in infant deaths, and compression and shifting trend of adult mortality, we were able to study the trend of the central part of the distribution of deaths in detail. In general, a right shift of the modal age at death for young adults is observed; in some cases, it is also accompanied by an increase in the number of deaths at these ages: in particular for France, in the last twenty years, premature mortality increases.

## Introduction

The general increase in life expectancy could lead to the conclusion that all people live longer. Vaupel et al. (2011) indeed showed that the longest life expectancies are observed in populations where lifespan variation is low. They also pointed out that the reduction in disparities is due to averting premature deaths. However, instead of observing a greater compression of mortality around the modal age at death, an increase in the variability is observed in some industrialized countries (Lynch and Brown 2001; Rothenberg et al. 1991). Moreover, premature mortality is strongly associated with health inequalities (Romeder and McWhinnie 1977), and lifespan disparity is higher and has been increasing faster among people with low education level (Van Raalte et al. 2011). The decrease in early mortality leads thus to greater longevity and greater equality between individuals, so the identification of the impact and the trend of premature deaths can be the first step to achieve the two goals.

What is premature mortality? How to recognize and measure it? The problem of identification of this component is tied to the difficulty of characterizing and separating it from adult mortality. According to different authors, several definitions have been proposed. For Eurostat, the term indicates all the deaths occurring before age 65, or the usual age at retirement (World Health Organization 2003). This definition can be useful and convenient because it leads to a clear separation between the two types of deaths. However, it cannot be employed to study premature mortality in the past or in countries where life expectancy at birth is less than (or close to) 65 years. Furthermore, this definition of retirement age is currently being adjusted to an increasing life expectancy at birth.

Two measures of life disparities are related to premature mortality: Year Life Lost (Murray et al. 2012) and dispersion in age at death, or $e^{\dagger }$ (Vaupel and Canudas-Romo 2003). Both of them are based on life expectancy at birth, so they give a concise measure of mortality inequalities, including information for all ages. Contrary, the approach here developed includes a full component solely on premature mortality: the identification of its distribution starting from the deaths curve, its mean, mode, standard deviation, skewness and their relation with other elements of the deaths distribution can be studied. Our parameterization further facilitates the calculation of the percentage of deaths due to premature mortality and the mean years lived for people dying prematurely.

There is a long tradition of authors publishing theories about mortality components and the possibility of recognizing them using the distribution of deaths (Barnett 1958; Benjamin 1959; Clarke 1950; Lexis 1879; Pearson 1897). The most famous approach was introduced by Lexis (1879), who divided the distribution into three parts: infant, premature and “normal” deaths. The first part starts at age 0 and finishes where the minimum, between ages 10–12, is encountered (Ebeling 2018). To determine the area of adult mortality, Lexis considered the shape of the death curve from the adult modal age to the last age at death and left-flipped it, in order to obtain a symmetrical section, which resembles a Gaussian normal distribution. These deaths under the Gaussian curve are referred to as normal deaths. Then, premature mortality designates the transition region between childhood and adult deaths. This identification of the adult modal age at death has been used to understand the development of mortality across the twentieth century (Bongaarts 2005; Cheung et al. 2005, 2009; Canudas-Romo 2008, 2010; Cheung and Robine 2007; Horiuchi et al. 2013; Kannisto 2000, 2001; Ouellette and Bourbeau 2011; Wilmoth and Horiuchi 1999; Wilmoth and Robine 2003). Pearson (1897) evaluated the problem from a statistical point of view: taking Lexis’ idea even further and considering the distribution of deaths to be composed of five functions with different degrees of skewness. In particular, he distinguished between infancy and childhood mortality and proposed for the first a negative exponential curve, covering also the antenatal period, and for the second a highly skewed distribution. Pearson also differentiated Lexis’ transitional region between youth (accidental) and middle life (premature) deaths. For both of these, he drew a normal distribution with one mode around age 25 and another around age 40. Finally, he identified old mortality like a skew distribution with skewness toward younger ages. Pearson justified his selection in two ways: (i) his theoretical argument was that the number of deaths at older ages must depend on the incidence of deaths at earlier ages, so it cannot be symmetrical, while (ii) the practical reason was that, without a skew curve, he was not able to obtain a satisfactory fit of the overall curve (Pearson 1897).

Even if the distribution of deaths by age is a good instrument to separate mortality components, generally, the models proposed in the literature based on death rates do not take into consideration premature mortality nor the accidental hump (Bennett 1983; Gompertz 1825; Kannisto 1994; Makeham 1860; Siler 1979; Weibull 1939). Heligman and Pollard (1980), based on the work of Thiele (1871), inserted three parameters to capture accidental mortality, but this component does not include premature mortality. Indeed, accidental and premature mortality are not the same thing: the first indicates deaths occurring around early adult years, which are usually identified as the “accidental hump” in the death distribution. This excess of mortality is observable mainly in human male distributions (Remund et al. 2018), and it is connected with sexual maturity, which depends on testosterone production (Parkes 1976), that increases the risk-taking behaviors. However, this increment is also related to socioeconomic vulnerability, as explained by Remund (2018), who showed how the favorable social context can reduce the risk of dying during these ages. Goldstein (2011) showed that, since 1750, the peak of the accidental hump has shifted to early ages from age 22 to age 18. The evolution of this trend depends on both nutritional status and disease environment. External causes of deaths, as suicide, homicide and accidents, are the main responsible of young adults deaths. Also the HIV/AIDS epidemic contributed to the peak in particular in the USA between 1980s and 1990s. The role of traffic and other accidents has decreased in the last few years (Remund et al. 2018).

On the other hand, premature mortality is a more wider concept. It describes all the deaths which take place before the “natural” age of deaths, although there is no consensus on the age threshold distinguishing deaths of old-age and premature ones. In statistical terms, premature mortality designates all the youth and young adulthood deaths happening outside the adult mortality area except infant and childhood deaths. The leading cause of death before old-age is neoplasm (Mazzuco et al. 2018b). According to a new classification of causes of death proposed by Camarda et al. (2015), there exists a group of degenerative diseases due in particular to strong man-made component, implying that an important part of these deaths can be preventable. Indeed, in the literature, the link between mortality and life styles is well known: for example, on average, people with a higher socioeconomic status live longer than others (Antonovsky 1967; Hattersley 1997; Huisman et al. 2004; Marmot and McDowall 1986; van Raalte et al. 2018). Another characteristic of premature mortality is that it produces life disparity: its complete postponement can reduce the entropy of the life table and increase the general life expectancy at birth. In other words, premature mortality designates all the youth and young adulthood deaths that occur outside the adult mortality area, which can be identified only by looking at the death curve. Although with drawbacks, the distribution of deaths has the advantage of being a density function, so it is possible to use a mixture of continuous probability distributions to approximate it. Strictly following Pearson’s approach, this corresponds to a model with no fewer than 13 parameters, with identification problems. The aim of this study is to work with a more parsimonious parametric model, which has the capacity to fit the entire age schedule of mortality, including a specific flexible function to model at the same time accidental and premature mortality. Even if accidental and premature mortality are different in distribution shape and position (and probably causes and mechanisms that generate their deaths), both produce early deaths, which are important to identify and detect on the whole to better understand the mortality evolution. The usefulness of this model to capture premature mortality is illustrated here by fitting the age distribution of deaths in several European countries.

The area of premature mortality partially overlaps the area of adult mortality, so that the two components seem to be a unique distribution. There is no visible breaking point or range of ages, which gives some indication of the position of this distribution. However, based on parameter estimates of the proposed model, the evolution of mortality in the middle part of the distribution of deaths by age can be analyzed. Our model uses several distributions, accounting for each of the components of the mortality age-profile. For all these distributions, it is possible to compute in explicit form mean, variance and skewness. Furthermore, it is even possible to distinguish between adult and the young modal ages at death.

This study is organized as follows: in Sect. 2 the data employed for the illustrations are described; Sect. 3 explains the method used to implement the model, and its advantages are discussed; in Sect. 4 results are shown; and Sect. 5 includes discussion and conclusion.

## Data

To fit the model, we analyze period death and exposure counts by single age and year from the Human Mortality Database (HMD) (Barbieri et al. 2015). We focus, in particular, on male populations from Sweden, France, East Germany and the Czech Republic. These populations are chosen to summarize mortality trends we observe for other European countries from the north, south, center and east of the continent. The reasons behind the choice of working only with male populations are twofold: (i) greater propensity to observe accidental mortality in their distribution of deaths; and (ii) a predisposition of asymmetry in the last part of the curve. Both elements are more challenging in the male than in the female populations, and this motivates our choice. For all of these populations, life tables were computed following standard procedures (Preston et al. 2001).

## Method

Historically, the death rates are the first choice to fit the age-patterns of mortality (Gompertz 1825; Makeham 1860; Weibull 1939; Siler 1979; Bennett 1983; Kannisto 1994). Heligman and Pollard (1980) specified an eight parameters model for the odds ratio of probability of dying that can fit accidental mortality. More recently, parametric and nonparametric models have been used to fit the mortality curve. For example, the CoDe model, proposed by De Beer and Janssen (2016), has 10 unknown values and it was specifically developed to study the mortality compression in youth, adult and advanced ages, as well as describing the full age pattern. De Beer and Janssen (2014) also introduced an additional generalization of the Heligman and Pollard model, which also includes 10 parameters. An additional work to mention corresponds to Basellini and Camarda (2016), who showed that the distribution of deaths can be employed to understand the transformations of mortality, in particular shifting and compression of adult deaths.

Gompertz, Makeham, Thiele and Heligman–Pollard proposed mathematical functions which take into account premature mortality. However, this concept is different in each model. For Gompertz and Makeham, it is something fixed across ages: Gompertz used the parameter *a* to describe the initial size of mortality, and Makeham added a constant representing deaths occurring randomly with respect to age. Both Thiele and Heligman–Pollard considered as premature mortality only the accidental hump. In our model, premature mortality is the sum of accidental mortality and the excess of deaths occurring before old-age. Moreover, it is modeled using a distribution, which is time and age variant.

The model used in this paper is inspired by Pearson’s idea on mortality components that distinguish between adult deaths and premature ones, and which fits all the age-distribution of deaths. A simplified version was introduced by Mazzuco et al. (2018a) to analyze mortality and to discuss the statistical advantages of working with a parametric approach. In particular, the authors point out that to approximate the characteristic shape of the deaths, density functions are required, the maximum likelihood is directly applied, and no constraints on the parameters and the function need to be set up. Moreover, it is possible to reconstruct the entire life table with the obtained modeled distribution of deaths $d_x$.

The model here used is a variation of the approach implemented by Mazzuco et al. (2018a), who proposed a mixture of one Half Normal and a Bimodal Skew Normal distribution (Elal-Olivero et al. 2009; Rocha et al. 2013) to fit the death curve. This method works in many contexts, and it is able to approximate several mortality paths, including excess mortality at young ages, for example due to HIV. The model has some restrictions about the values that the coefficients regarding premature mortality can assume, since the Bimodal Skew Normal can be seen as a mixture of two functions with fixed values. To obtain a better flexibility and study more specifically the evolution of the death curve in its middle part, these restrictions are eliminated and a mixture of three distributions is adopted (for more details, see “Appendix A”). Infant mortality and child mortality are summarized employing a Half Normal distribution, which is defined only for values greater than 0. Moreover, it is possible to use its mode like a measure of infant mortality level. This distribution has the following probability density function:

1 $$\begin{aligned} f_I(x) = \frac{\sqrt{2}}{\sqrt{\pi }}\exp \left( -\frac{x^2}{2} \right) \quad x>0, \end{aligned}$$

where *x* is the age at death. Different from Mazzuco et al. (2018a), the $\sigma$ parameter is set to 1 and thus omitted here; furthermore, this value allows fitting infant mortality and the decline of the curve after infancy, avoiding identification issues.

A Skew Normal distribution (Azzalini 1985) is adopted for adult mortality. This class of distributions includes the normal one as a particular case, so it is possible to control if the adult distribution requires an asymmetrical function, in line with Pearson’s theory, or if a symmetrical one is sufficient. The distribution selected to model and combine the accidental and premature mortality follows the shape of the death curve in its middle part. Certainly modeling accidental and premature component separately would have been theoretically the best choice, but practically impossible: an additional distribution leads to more identification problems. Moreover, our target is to present a new model which can separate early deaths (accidental and premature together) from adult mortality, which is historically considered as the group of premature and senescent deaths together and then fitted with a single function. By subtracting the infant, child and adult components of the distribution of deaths, an asymmetrical shape of residuals is observed. Moreover, the new distribution should fit the accidental hump when it is reasonably visible, without losing the fit for premature mortality. For these reasons, another Skew Normal distribution is employed: its flexibility allows us to capture accidental mortality without losing premature deaths. Thus, the distribution is a compromise between the two symmetrical curves Pearson described. The idea of using an asymmetrical distribution to fit the middle part of the curve was already proposed by Kostaki (1992), who modified the Heligman–Pollard model in order to obtain better estimates. However, the author considered only the excess deaths due to the accidental hump, while in our approach both accidental and premature components are modeled together with a skew function. For accidental and premature mortality, and for adult mortality, we have the following functions, respectively:

2 $$\begin{aligned} f_{m}(x;\theta _m)= & {} \frac{2}{\omega _m}\phi \left( \frac{x-\xi _m}{\omega _m}\right) \Phi \left( \lambda _m \frac{x-\xi _m}{\omega _m}\right) , \end{aligned}$$

3 $$\begin{aligned} f_{M}(x; \theta _M)= \frac{2}{\omega _M}\phi \left( \frac{x-\xi _M}{\omega _M}\right) \Phi \left( \lambda _M \frac{x-\xi _M}{\omega _M}\right) , \end{aligned}$$

where $\phi (\cdot )$ is the standard normal probability distribution function, $\Phi (\cdot )$ the standard normal cumulative distribution function, *m* indicates the formula for accidental and premature mortality, while *M* the one for adult component. Each distribution has three parameters, $\theta _m = (\xi _m, \omega _m, \lambda _m)$ and $\theta _M = (\xi _M, \omega _M, \lambda _M)$, where $\xi _{(\cdot )} \in \mathbb {R}$ corresponds to the location, $\omega _{(\cdot )} \in \mathbb {R}^+$ for the scale and $\lambda _{(\cdot )} \in \mathbb {R}$ for the skewness. If $\lambda _{(\cdot )} =0$, a Standard Normal density function is obtained.

**Fig. 1:**
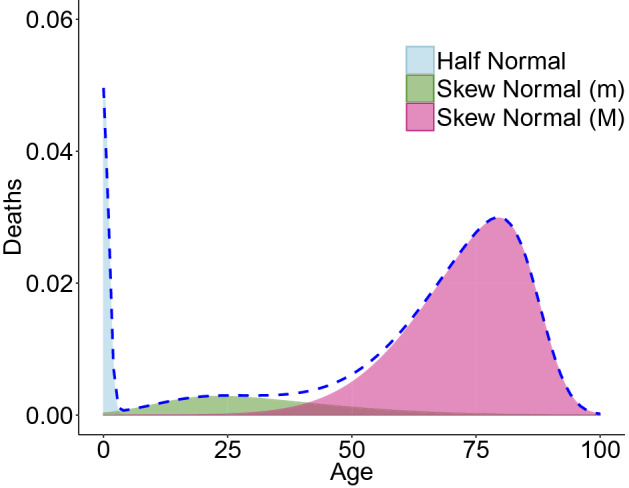
Stylized distribution of death (dotted line) and the three functions of the mixture model

Combining Eqs. (1), (2) and (3) with the mixture (or weighting) parameters $\eta$ and $\alpha$, a model with eight coefficients is obtained (see Fig. 1):

4 $$\begin{aligned} f(x;\,\theta ) = \eta \cdot f_I(x) + (1-\eta ) \cdot \Big [ \alpha f_{m}(x; \theta _m) \; + (1-\alpha )f_{M}(x; \,\theta _M) \Big ], \end{aligned}$$

where $\theta$ is the vector of 8 parameters, $\eta$ is the first mixture parameter with value ranging in [0, 1], and $\alpha$ is the second mixture parameter which also varies in the interval [0, 1]. Equation (4) is a generalization of the model proposed by Mazzuco et al. (2018a), which permits identification of the premature mortality component, as shown in Sect. 4.2.

Mazzuco et al. (2018a) tested the goodness of fit of their model, by comparing it with Heligman and Pollard, and Siler models, since both are used to approximate the entire age schedule of mortality. Unlike the Siler, their model is able to capture extra mortality at young ages and it is more parsimonious than Heligman and Pollard, which often has the problem of overparameterization (Congdon 1993). The existence of identification issues caused by the correlation of the coefficients present in particular between Siler and Heligman and Pollard’s parameters was also shown. Actually, the Binomial Skew Normal distribution is itself a mixture of two Skew Normals, with some restriction on the parameters values, so model (4) presented here is a generalization of the model of Mazzuco et al. (2018a). Thus, their results hold also for our method, except for the fact that the number of parameters increased and identification problems can be an issue during the estimation process. Ways to overcome this limitation are discussed in Sect. 3.3.

### Demographic Interpretation of the Parameters

The first mixture parameter $\eta$ is the intensity of infant mortality, and it is related to the probability of dying in the first year of life, $_{1}q_0$. Moreover, this value is also associated with the variance of the first part of the distribution of deaths, which explains how quickly child mortality decreases. Considering Eq. (1), its variance is:

5 $$\begin{aligned} {\hbox {Var}}(\eta f_I)=\eta ^2\left( 1 - \frac{2}{\pi }\right) , \end{aligned}$$

which depends only on the parameter $\eta$. In Eq. (4), the second mixture coefficient is $\alpha$, which indicates the importance of the premature mortality (with 0 for the case without premature mortality).

To better understand the role of the parameters of $f_m$ and $f_M$ functions, it is useful to rewrite the coefficients in terms of mean, variance and skewness. This type of mathematical calculation is called centered parametrizations, and it is also convenient in the estimation process, as explained in Sect. 3.3. The details of the re-parametrization are reported in “Appendix B”.

The three parameters of $f_m$ are: $\mu _m$ is the mean and it is associated with the position of the mode of accidental and premature mortality; $\sigma _m$ is the variance of the distribution, so if its value is small the premature mortality is concentrated at few ages, while if its value is big, we obtain a very flat function (with premature deaths present in a wide age interval); if the third parameter $\gamma _m$ is positive, we obtain a skewness on the right; otherwise, the skewness is on the left.

There are also three parameters for $f_M$: $\mu _M$, is the average of adult mortality and it is related to the main modal age at death; $\sigma _M$ corresponds to how much the adult deaths are concentrated around the adult mode and it can be seen like a measure of adult mortality compression; $\gamma _M$ is the parameter of skewness. The latter allows us to verify Pearson’s theory on the skewness of adult death distribution. A value significantly different from 0 means that adult deaths have a skew distribution. In particular, the parameter is expected to have negative values because, usually, the adult distribution of deaths shows an asymmetry toward young ages (left).

An important measure of longevity used to understand mortality changes is the old modal age at death (Bergeron-Boucher et al. 2015; Canudas-Romo 2008; Cheung et al. 2005; Horiuchi et al. 2013; Missov et al. 2015). Model (4) identifies three different modes: *I* related to infant mortality, *m* for accidental and premature component, and *M* the adult modal age at death. The Half Normal distribution, describing infancy and childhood mortality, always has its mode at age 0, while the others are related to the two Skew Normals, and numerical computation is required to identify them.

It is also possible to split the area under the distribution of deaths into three parts (see Fig. 1). Each area corresponds to the percentage of deaths in the infancy and childhood, accidental and premature, and adult mortality. For example, the infant and childhood mortality area ($A_I$) can be measured with the integral:

6 $$\begin{aligned} A_I = \int _{0}^{\Omega } \eta \cdot f_I(0;1) \; {\hbox {d}}x = \eta , \end{aligned}$$

where $\Omega$ is the highest attained age at death. In fact, we can assume that the Half Normal distribution spreads its probability in all the intervals $[0,\Omega ]$. Similar calculations can be done for accidental and premature mortality area ($A_m$) and adult mortality area ($A_M$) (see Table 1).

**Table 1:** The areas for the different components of the deaths distributions

Mortality	Area
Infant	$A_I=\eta$
Premature	$A_m = \alpha (1-\eta )$
Adult	$A_M = (1-\eta ) (1-\alpha )$

### Life Expectancy Decomposition

An attractive feature for a mortality model is the possibility to compute in explicit form the life expectancy at birth (Missov 2013; Missov and Lenart 2013; Vaupel and Missov 2014). The mixture approach allows not only to compute $e_0$ analytically, but also to decompose the contribution to life expectancy of the three different components: infant and childhood, accidental and premature, and adult mortality.

Indeed, life expectancy at birth, $e_0$, is the mean age of the distribution and it can be decomposed as:

7 $$\begin{aligned} e_0&= \int _{0}^{\Omega } x \cdot f(x,\theta )\; {\hbox {d}}x \nonumber \\&= \; \eta \left( \frac{\sqrt{2}}{\sqrt{\pi }}\right) + (1-\eta ) \alpha \left( \xi _m + \omega _m\frac{\lambda _m}{\sqrt{1+\lambda _m^2}}\sqrt{\frac{2}{\pi }}\right) \nonumber \\&\quad +(1-\eta ) (1-\alpha ) \left( \xi _M + \omega _M\frac{\lambda _M}{\sqrt{1+\lambda _M^2}}\sqrt{\frac{2}{\pi }}\right) \nonumber \\&= e_I + e_m + e_M, \end{aligned}$$

corresponding to the each of the three means of the model functions multiplying their appropriate mixture parameters (more details concerning the calculation of Eq. 7 can be found in “Appendix C”).

The overall $e_0$ is the sum of the single average ages at death of the three components of mortality in the model, weighted by their mixture parameters $\eta$ and $\alpha$. Equation (7) needs to be interpreted as the mean years lived for those dying in the different age groups: $e_I$ is the average age at death by those dying during infancy and childhood, $e_m$ the mean number of years lived by those dying in middle life, $e_M$ is the average years duration for those dying in adulthood. As shown in the results section, in low-mortality populations, life expectancy at birth and the average age at death at adult ages are practically the same $e_0 \approx e_M$.

### Estimation of the Model Parameters

We use maximum likelihood to estimate the parameters of the mixture model. The data available are in aggregate form: we do not know the exact age of death for every individual, but the number of deaths in every age interval. The intervals are disjointed and mutually exclusive (individuals die only once) and space partitioned (they cover all the life span). Therefore, since we are modeling the probability of the number of deaths that occur in the age interval $(x,x+1)$, the multinomial distribution is appropriate (Azzalini 2017). Thus, the likelihood function that follows is:

8 $$\begin{aligned} L(\theta ;\,D_x)= \prod _{x=0}^{\Omega }p(x;\,\theta )^{D_x}, \end{aligned}$$

where $D_x$ are the real death counts at age *x* and $p(x;\theta )$ corresponds to the probability of dying in the interval *x* and $x+1$, which can be computed as the integral of the mixture model between two ages:

9 $$\begin{aligned} p(x;\,\theta )=\int _{x}^{x+1} f(t;\,\theta ) \; {\hbox {d}}t. \end{aligned}$$

**Fig. 2:**
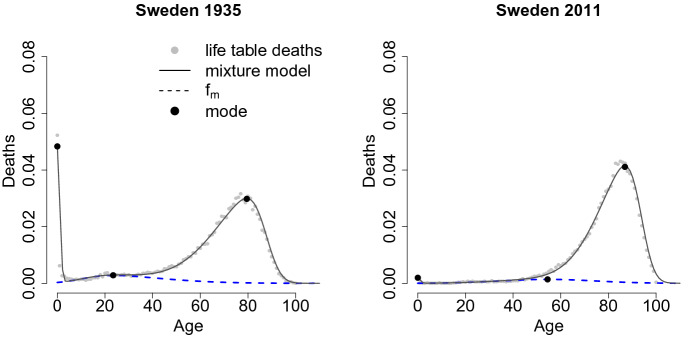
Model fit on life table deaths for Sweden in 1935 and 2011. The solid line shows the overall mixture model. The dotted line highlights the fit of the Skew Normal employed to estimate accidental and premature mortality. The big dots point out the three modal ages of the distribution

Maximizing Eq. (8), the parameter values are obtained. To guarantee more stable estimates for the coefficients, the centered parametrization is used, instead of the direct one. Indeed, because of the shape of the likelihood, a local maximum is often chosen instead of the global one (Azzalini and Capitanio 1999). The re-parametrization reported in “Appendix B” allows a more regular shape of Eq. (8) and also more suitable results. As an example, Fig. 2 shows the fitted model for Swedish data for two different years (1935 and 2011).

As seen in Fig. 2, in each year the estimated model is close to the real data points and it is smooth enough, so that it clearly shows the trend of the life table distribution of deaths. Considering all the estimated countries, the mixture function indicates a good approximation in terms of errors: the sum of the absolute value of the differences between model estimates and input data returns a median error below 0.05.

For the estimation of the coefficients the maximization of the likelihood function (8) is required. To reduce the risk to find a local maximum instead of the global one, we selected the algorithm DEoptim implemented in R, which is particular appropriate when there is the suspicion of local maxima (Mullen et al. 2011). The optimization algorithm, instead of using a single vector as starting point, it specifies a matrix, in which each row represents a coefficient. The initial population is generated randomly within the lower and upper boundaries. The algorithm starting from the different combination of parameters, estimates the likelihood. The one with higher values is then selected. To reduce the risk of failure, 1000 iterations are set for the parameter estimations each year. If the final estimation was not satisfactory, we restarted the algorithm including more random combinations and iterations. Then, to maximize the likelihood nlminb was selected which leads to close results obtained with DEoptim, but it is more efficient (computation time is lower).

In “Appendix D”, as an example, a shred of the code is reported to estimate the parameters of the model for Sweden 2009. The functions to fit the mixture mode are also publicly available on GitHub.

To detect the range of the parameter values and study the errors due to the estimation process, some simulations were performed. Four different patterns of death distribution were chosen (Sweden 1930, France 1944, 1990 and 2010). For each of them, the corresponding life tables were computed using the vectors of parameters estimated. 1000 sets of random values for the eight coefficients were generated using Uniform distributions with their support delimited by the range of parameters. The maximum likelihood was computed with nlminb algorithm inserting as starting point each random vector and considering the $d_x$ calculated previously. The estimates obtained were compared with the original set of parameters. All these results are reported in Sect. 4.5.

To estimate the significance of the parameters, we need to compute their standard errors. Thought computationally intensive, bootstrap techniques (Efron 1979) allow to recreate the distribution of the coefficients and their values. In particular, it is interesting to study the role of the Skew Normal to fit premature mortality, in order to detect if its contribution to the model is really indispensable. To answer the question, we can look at the significance of the mixture parameter $\alpha$, which indicates the importance of this component in the overall mortality (if $\alpha$ is 0, the middle component is automatically deleted, and the model can be reduced to a mixture of one Half Normal and only one Skew Normal). Bootstrap was applied to the same cases used for simulations. The results are reported and discussed in Sect. 4.4.

## Results

### Infant, Child and Adult Mortality

**Fig. 3:**
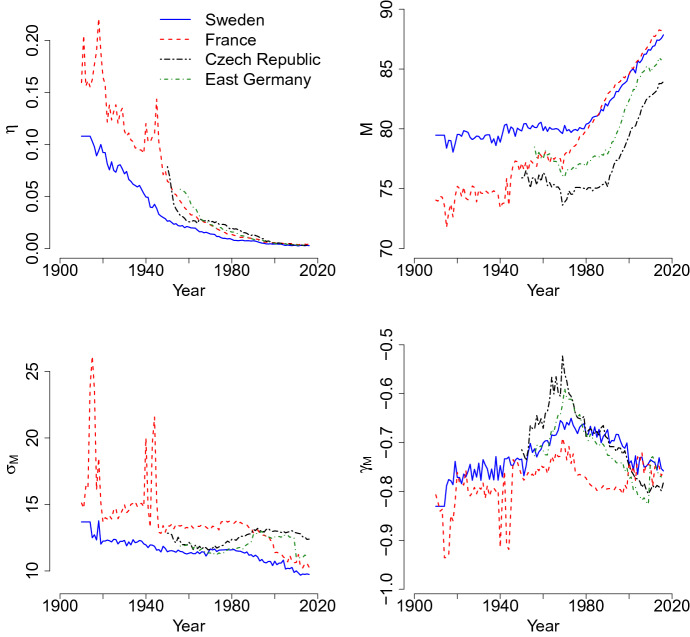
Time trends of the mixture parameter associated with infancy and childhood mortality $\eta$, and of the adult mortality components: mode *M*, variance $\sigma _M$ and skewness $\gamma _M$

As shown in Fig. 3, the trends of the coefficients of infant, child and adult mortality confirm the known tendencies. During the demographic transition, most developed countries experienced a reduction in infant mortality (Edwards and Tuljapurkar 2005; Vaupel et al. 2011; Wilmoth and Horiuchi 1999). The incidence of deaths at age 0 decreases, as seen in the decrease over time in the estimated mixture parameter $\eta$. Figure 3 further shows that for all the populations in recent years, $\eta$ is very close to 0, which means that infant mortality is very small.

During the first half of the twentieth century, in low-mortality countries, a compression in a smaller age interval of the adult mortality distribution was observed (Cheung et al. 2005, 2008, 2009; Cheung and Robine 2007; Fries 1983; Kannisto 2001; Wilmoth and Horiuchi 1999). After a period of strong compression, developed countries experienced a shift of the late modal age at death (Bongaarts 2005; Canudas-Romo 2008; Cheung and Robine 2007; Kannisto 1996). This transition is observed also in the mixture model. As seen in Fig. 3, compression and shifting can be studied using the values of the variance $\sigma _M$, and the trend of the mode *M* (related to the value of the mean $\mu _M$), respectively. For Sweden and France, which have a longer time series, the compression of the adult distribution is seen by the reduction in $\sigma _M$. The other two populations present almost invariant values of $\sigma _M$, with a slight increment since 1980–1985. In Fig. 3, for all the populations, an increase in the mode *M* is reported. This means that the late modal age at death progressively shifts to the right of the distribution of deaths. Thus, parameter estimates are consistent with the previous literature. It is interesting to note that the value of $\gamma _M$ is quite stable in the observed period, with a peak occurring in 1950–1980. This means that the left asymmetry of the adult mortality is a stable feature of this component. The irregularities observable for France, for the coefficient $\eta$, the variance and the index of symmetry coincide with the years of the two world wars, and are also perceived in the other model coefficients.

### Premature Mortality

**Fig. 4:**
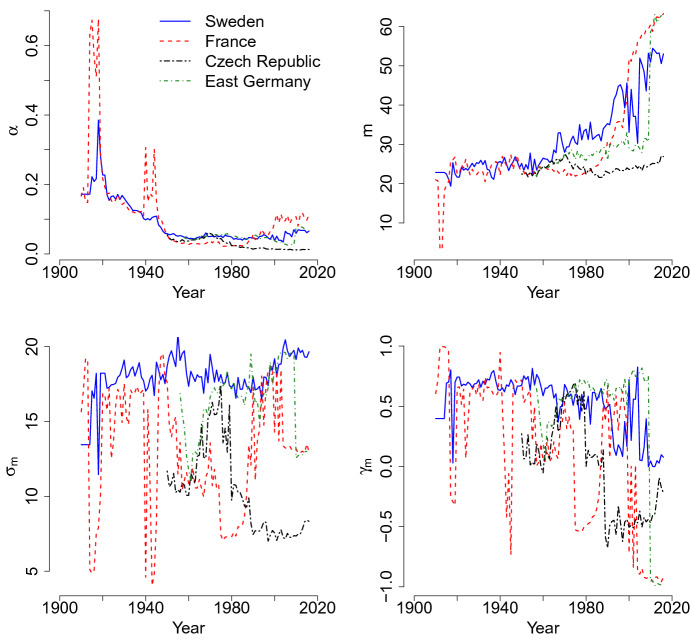
Trends over time of the mixture parameter $\alpha$ and of accidental and premature mortality components: mode *m*, variance $\sigma _m$ and skewness $\gamma _m$

In Fig. 4, the coefficients of the premature mortality of the $f_m$ distribution are presented. For Sweden, France and East Germany, an increase is seen in the mode of premature deaths *m*, with a particular acceleration trend starting in the last 20 years of the twentieth century. The increment is particularly evident in France. Moreover, for these populations, the range of variation is very similar, except for the Czech Republic, which has a quite constant mode around age 25.

The parameter related to the skewness is $\gamma _m$. As seen in Fig. 4 in most of the cases, its values is positive, but close to 0 suggesting that the curve is almost symmetrical.

For the variance $\sigma _m$ (see Fig. 4), the estimates are affected by identification problems. However, it is possible to seize different time trends. For Sweden and East Germany, the values are quite stable around 15–20, meaning that the shape of accidental and premature mortality is quite stable in the period, also considering that $\gamma _M$ has the same pattern. Also France seems to follow this tendency except for 1950–1980 and the years of the world wars, where an increase in mortality in the middle part of the distribution of deaths is registered. In the Czech Republic, $\sigma _m$, after a peak between 1950 and 1980, the term decreases because the distribution becomes more concentrated around the accidental mode.

Finally, in Fig. 4, we consider the trend of the mixture parameter $\alpha$, which is related to the incidence of accidental and premature mortality in the overall distribution of deaths. Both in Sweden and France, the parameter decreases until 1990. In France, two peaks are observed in correspondence of the two world wars: in those years, the number of young deaths increases. For these two countries, the general declining trend of $\alpha$ is related to the disappearance of male accidental hump and the compression of adult mortality around the late mode at death. Recently, especially in France, an increase in $\alpha$ is registered, which means that premature mortality acquires relevance.

### Life Expectancy Components

**Fig. 5:**
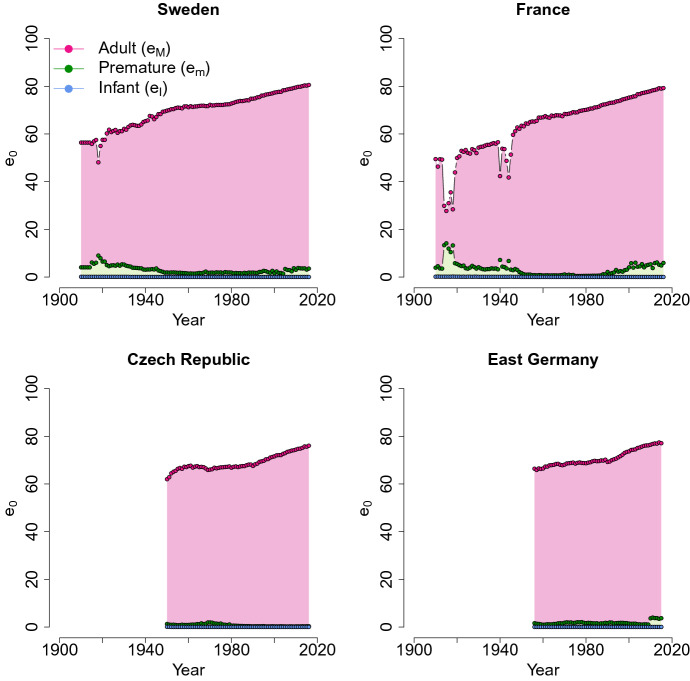
The trend of life expectancy at birth decomposed into the three mortality components. For each year, the amount of every part is identified by a different color. The overall level ($e_0$) is given by the sum of the contributions of the infant, accidental and premature, and adult mortality

The decomposition of $e_0$ for all the considered populations is shown in Fig. 5. In the graphs, we can see the contribution of the three components of the model, for infant and child part $e_{I}$, for accidental and premature mortality $e_{m}$ and for adult deaths $e_{M}$. In the Czech Republic and in Germany, the contribution of $e_m$ is very small—almost negligible—in particular during the recent years. In Sweden and France, its contribution reduced between 1930 and 1950 (except in France during the world war years), and then it became constant, without disappearing. In the last few years (1990–2011), the premature mortality component increased to capture the deaths occurring in the central part of the curve.

### Significance of the Parameters

To test the significance of the parameters, we selected five different cases: Sweden 1930, where infant mortality is high and also premature deaths are relevant for young and young adults; France during the Second World War (1944), that shows an excess of deaths between age 18 and 40, France 1990 which has a very low value for the parameter of premature mortality, France 2010, that is the emblematic case of the upswing of mortality before senescent. The standard errors to perform the test are computed by bootstrap using 1000 samples, whose numerosity depends to the number of deaths registered each year of the considered periods. In each scenario, all the coefficients are statistically significant with a $p \text{ value }<0.0001$ (standard errors are reported in Table 2).

**Table 2:** Standard errors computed by bootstrap techniques for the eight parameters of each scenario

	Sweden 1930	France 1944	France 1990	France 2010
$\sigma _m$	0.148	0.049	0.300	0.108
$\sigma _M$	0.056	0.055	0.047	0.091
$\alpha$	0.015	0.010	0.030	0.040
$\gamma _m$	0.014	0.028	0.027	0.013
$\gamma _M$	0.006	0.002	0.004	0.008
$\mu _m$	0.167	0.054	0.386	0.369
$\mu _M$	0.059	0.078	0.053	0.103
$\eta$	0.013	0.010	0.031	0.046

Since the value of the mixture parameter $\alpha$ is always different from 0, the distribution $f_m$ cannot be neglected. This means that even if the role of premature mortality is small (as, for example, in France 1990), it has to be consider to obtain a satisfactory fit of the overall curve. Indeed, without this component, all the deaths of adolescents, young and young adults are not approximated by the model and, in particular, the left side of the adult hump is not fitted adequately.

### Quality of the Estimates

The mixture model in (4) is a complex function, so the estimation of its parameters can be problematic because of possible local maxima and identification issues. Simulations were performed to detect the errors that may occur considering several random vectors of starting points. We use parameter estimates for the three cases considered in Sect. 4.4 (Sweden 1930, and France 1944, 1990 and 2010) to obtain four age distributions of deaths. For each scenario, the rescaled bias between the real coefficients and the ones estimated using the 1000 casual starting points are calculated:

$$\begin{aligned} {\hbox {RE}}_i = \frac{\theta - \hat{\theta _i}}{\theta }. \end{aligned}$$

**Fig. 6:**
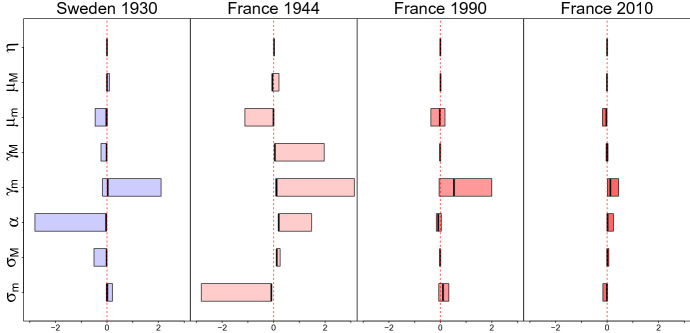
The 25th and 75th percentiles of bias distribution of each parameter considering four different scenarios. The ticker black line represents the median

In Fig. 6, the results obtained are summarized considering the 25th and the 75th percentiles of the errors distribution of each parameter. The main problem of this model can be found in estimating $f_m$ and its mixture coefficient $\alpha$: in several cases the median is not centered on the real value of the parameter and the distribution has heavy tails. The more problematic coefficient is $\gamma _m$, which has bigger ranges in each scenario. The Skew Normal employed for adult mortality, $f_M$, and its related mixture parameter $\eta$ are not particularly affected by the choice of the starting point: in most cases the bias distribution is concentrated around 0. Again the shape parameter $\gamma _M$ turns out to be the most affected by identification problems, even though, in this case, these issues are only limited to France 1944. Regarding the $f_m$ parameters, it was observed that the overall shape of the mixture curve is quite stable (also in France 1944). In general, in some cases there might be identification and local maxima issues, given by the complexity of the function, so considering that a numeric optimization algorithm should be used to maximize the likelihood in Eq. (8), starting values of parameters (especially of $\alpha , \mu _m, \sigma _m, \gamma _m$) should be chosen carefully.

## Discussion

A new parametric model to fit the life table distribution of deaths was proposed. This model is based on a mixture of a Half Normal distribution and two Skew Normal distributions. These functions were chosen to re-elaborate Pearson’s theory of mortality components. The Half Normal is used to fit infant and childhood mortality, one Skew Normal for accidental and premature mortality, and the second Skew Normal for adult mortality. The latter function allows adult mortality to be modeled with an asymmetrical distribution. The new model allows differentiation between premature and adult mortality. This differentiation is not often taken into account in the analysis of mortality, both because there is not clear definition of premature deaths and because the frequently used mortality models do not yield a separation between the two. In our alternative definition of accidental and premature mortality, instead of defining them as a consequence of the other two components (remaining part of the infant and adult model fitting), in the newly introduced model they have their own distribution. In this way, they have an unambiguous definition. Taking advantage of this, the trend and the transformations of accidental and premature mortality during the last century were analyzed, discovering differences among the observed populations.

All parameters of the model have a demographic interpretation, and they can be studied to analyze the characteristics and the transformations of mortality components. The results obtained for infant and childhood mortality show a reduction and a concentration of the incidence of deaths at age 0: the risk of dying after birth is very small, and the mortality during childhood has almost disappeared. For adult mortality, a general shift in the late mode to the right of the distribution was found. The conclusions about infant, childhood and adult mortality are consistent with what is already known about the trends of these components (Bongaarts 2005; Canudas-Romo 2008; Cheung et al. 2005, 2008, 2009; Edwards and Tuljapurkar 2005; Fries 1983; Kannisto 2001; Vaupel et al. 2011; Willets 2004; Yashin et al. 2001).

During the last century, the accidental hump disappeared for most of the countries, but the premature deaths across youth and the first part of adulthood continue to exist, even if with a small incidence (greater flattening of the deaths distribution in its middle part). It was observed that the populations undergoing a compression and then a shift in adult mortality show a right shift of premature mortality distribution toward older ages (ages 50–60). This means that accidental mortality has almost disappeared. However, for recent years, an increase in premature mortality due to deaths that occur near, but outside the adult distribution was observed. In fact, premature mortality has changed: in the past, it was identified with deaths around age 40. Now premature mortality is shifting to the right of the distribution of deaths, following the shift seen in adult mortality. In countries such as France and Sweden, which underwent a strong compression and a shift in adult mortality, we observed the disappearance of accidental mortality and an increase in premature deaths. For the nations where the adult mortality did not undergo a compression, like the Czech Republic and East Germany, the incidence of premature mortality is very small and sometimes the accidental hump is still present. This consideration suggests that the trend in premature deaths is correlated with that of adult mortality. In particular, for countries which show an almost parallel shift in the survival curve (Lynch and Brown 2001; Horiuchi and Wilmoth 1997, 1998; Robine 2001; Yashin et al. 2001), an increase in the number of deaths that occur outside the adult distribution was found. This phenomenon is clearly visible for France, which presents—simultaneously with the shift in the survival curve—an increase in the number of deaths related to premature mortality. This is consistent with the rise of lifespan variance recognized and illustrated by Engelman et al. (2014).

Existing relations between external causes of death (suicide, homicide and accidents) and premature mortality were further investigated, but no such relations were found. Perhaps the observed increment may be related to another disease (or a group of them) or to conditions associated with social and economic deprivation (lower education, unskilled occupation, etc.). For example, several authors have addressed the strong correlations existing between mortality and educational levels (Hattersley 1997; Huisman et al. 2004; Dalstra et al. 2006; Marmot and McDowall 1986; Shkolnikov et al. 2011; Strand et al. 2010; Valkonen and Tapani 2001; Zarulli et al. 2013, 2012) or health status for example, obesity (Olshansky et al. 2005). However, these are hypotheses of the reasons of the changes in premature mortality that need to be verified and further studied. A further possibility is that the premature mortality that is captured by our model is purely a statistical artifact of the residual part not captured by the infant, child and adult components. By estimating all the distributions at the same time, that possibility was greatly reduced. Further studying this possibility is beyond the scope of the current work. Premature mortality remains a puzzle for demographers, epidemiologists and other population health researchers.

In conclusion, in this article, a new mixture model for the distribution of deaths, and its relevance for studying accidental and premature mortality were shown. The latter two components are not usually taken into account due to the complexity involved in recognizing and separating them from adult mortality. The newly introduced method is useful to analyze contexts in which accidental and premature components play a relevant role in mortality. However, the model is not limited to this use, and researchers could apply it to study other aspects of the age-patterns and trends in mortality.

## Figures and Tables

**Fig. 7:**
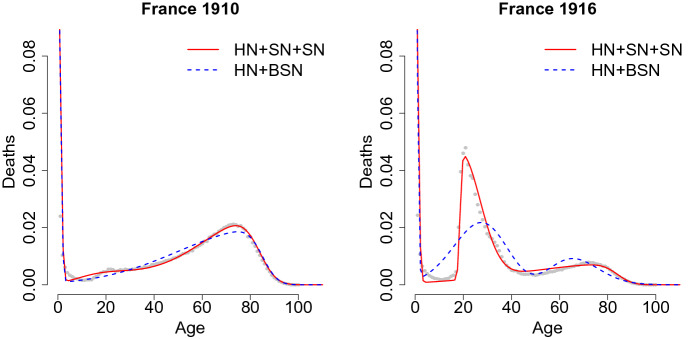
Comparison between the mixture model here suggested (one Half Normal and two Skew Normals) and model introduced by Mazzuco et al. (2018a) (one Half Normal and a Bimodal Skew Normal)

## Appendix A

This model is inspired by the one proposed by Mazzuco et al. (2018a), and it is based on a mixture three distributions in order to re-elaborate Pearson’s theory of mortality components. In Mazzuco et al. (2018a), a Bimodal Skew Normal distribution fits the second part of the death curve. This function is a mixture of two Skew Normals distributions having the same values of shape, location and scale parameters. This implies that the shape of premature mortality is the same as that of the adult mortality, and the only difference is its position and weight which is modified by the mixture parameter $\alpha$. An exemplification of this constraint is presented in Fig. 7. In Mazzuco et al. (2018a), the Skew Bimodal Normal (SBN) distribution was used because it provides a good fit with a relatively few number of parameters (only 6). In this paper, we focused more on premature mortality, so we needed more flexibility and interpretability of the coefficients. The generalization we proposed allows to better characterize the distribution of premature mortality and interpreter $\alpha$ as the importance of premature death, which was not possible with the SBN distribution.

## Appendix B

As explained in Azzalini and Capitanio (1999), the maximization of the likelihood function can be problematic when the direct parametrization, $DR(\xi ,\omega ,\lambda )$ is employed. In particular, when $\lambda =0$, there is always an inflection in the profile log-likelihood. Moreover, even when $\lambda$ is not close to 0, the shape of the likelihood is bimodal, so the global maximum is not easily identifiable. To reduce this issue, Azzalini proposed a different parametrization, which allows a more regular shape of the function. Instead of using $\xi , \omega$ and $\lambda$, the centered parameters are estimated, $CP(\mu , \sigma , \gamma )$, as the mean, variance and index of skewness, respectively. To go from $CP(\mu , \sigma , \gamma )$ to $DR(\xi ,\omega ,\lambda )$, the following equations are required

10 $$\begin{aligned} c&= {{\,\mathrm{sgn}\,}}(\gamma ) \left( \frac{2\gamma }{4-\pi }\right) ^{1/3}\nonumber \\ \mu _z&= \frac{c}{\sqrt{1+c^2}} \nonumber \\ \xi&= \mu - \frac{\sigma }{\sqrt{1-\mu _z^2}} \frac{c}{\sqrt{1+c^2}} \end{aligned}$$

11 $$\begin{aligned} \omega&= \frac{\sigma }{\sqrt{1-\mu _z^2}} \end{aligned}$$

12 $$\begin{aligned} \lambda&= \frac{\mu _z \sqrt{\pi /2}}{\sqrt{1-\frac{\pi \mu _z^2}{2}}}. \end{aligned}$$

The inverse procedure is described in Arellano-Valle and Azzalini (2008). The centered parametrization is always used to estimate the parameters.

## Appendix C

Life expectancy at birth can be computed as the mean age at death using equation (4):

13 $$\begin{aligned} e_0&= \int _{0}^{\Omega } x \cdot f(x,\theta )\; {\hbox {d}}x \nonumber \\&= \int _{0}^{\Omega } x \cdot \Big \{\eta \; f_I(x;\,1) + (1-\eta ) \cdot \Big [ \alpha f_{m}(x;\, \theta _m) \; + (1-\alpha )f_{M}(x;\, \theta _M) \Big ] \Big \} \; {\hbox {d}}x\nonumber \\&= \int _{0}^{\Omega } \eta \; x f_I(x;\,1) \; {\hbox {d}}x+ \int _{0}^{\Omega } (1-\eta ) \alpha \; x f_{m}(x;\, \theta _m) \; {\hbox {d}}x \nonumber \\&\quad +\int _{0}^{\Omega } (1-\eta ) (1-\alpha ) \; x f_{M}(x;\, \theta _M) \; {\hbox {d}}x \nonumber \\&= \eta \int _{0}^{\Omega } x f_I(x;\,1) \; {\hbox {d}}x+ (1-\eta ) \alpha \int _{0}^{\Omega } x f_{m}(x;\, \theta _m) \; {\hbox {d}}x \nonumber \\&\quad +(1-\eta ) (1-\alpha ) \int _{0}^{\Omega } x f_{M}(x;\, \theta _M) \; {\hbox {d}}x, \end{aligned}$$

which can be split into the single means of the three distributions involved, multiplied by their mixture parameters. Since $\sigma =1$, the mean of the Half Normal distribution is given by:

14 $$\begin{aligned} E_I[X]=\int _{0}^{\Omega } x f_I(x;\,1) \; {\hbox {d}}x = \frac{\sqrt{2}}{\sqrt{\pi }}, \end{aligned}$$

while the expected value for the Skew Normal distribution is provided by Azzalini and Capitanio (1999):

15 $$\begin{aligned} E_{m}[X]&= \int _{0}^{\Omega } x f_{m}(x;\, \theta _{m}) \; {\hbox {d}}x = \left( \xi _{m} + \omega _{m} \frac{\lambda _{m} }{\sqrt{1+\lambda _{m} ^2}}\sqrt{\frac{2}{\pi }}\right) , \end{aligned}$$

16 $$\begin{aligned} E_{M}[X]&= \int _{0}^{\Omega } x f_{M}(x;\, \theta _{M}) \; {\hbox {d}}x = \left( \xi _{M} + \omega _{M} \frac{\lambda _{M} }{\sqrt{1+\lambda _{M} ^2}}\sqrt{\frac{2}{\pi }}\right) . \end{aligned}$$

Substituting Eqs. (14), (15) and (16) in Eq. (13), the decomposition of life expectancy at birth is obtained (Eq. (7)).

## Appendix D

The R code to obtain the probability density function and the likelihood of the mixture model (4) is shown below.

Here an example is reported to estimate the parameters of the mixture distribution for Sweden 2009. The data employed are the male life table with 1-year age and calendar time intervals, computed by the Human Mortality Database.
